# MPP2 interacts with SK2 to rescue the excitability of glutamatergic neurons in the BLA and facilitate the extinction of conditioned fear in mice

**DOI:** 10.1111/cns.14362

**Published:** 2023-07-19

**Authors:** Xiaohan Peng, Panpan Chen, Yang Zhang, Ke Wu, Ningning Ji, Jinghua Gao, Hui Wang, Yong‐mei Zhang, Tie Xu, Rong Hua

**Affiliations:** ^1^ NMPA Key Laboratory for Research and Evaluation of Narcotic and Psychotropic Drugs Xuzhou Medical University Xuzhou China; ^2^ Jiangsu Province Key Laboratory of Anesthesiology Xuzhou Medical University Xuzhou China; ^3^ Anesthesiology Department Jiangsu Province Hospital Nanjing China; ^4^ Emergency Medicine Department The Affiliated Hospital of Xuzhou Medical University Xuzhou China

**Keywords:** basolateral amygdala, membrane palmitoylated protein2, PKA, PTSD, SK2 channels

## Abstract

**Aims:**

The basolateral amygdala (BLA) plays an integral role in anxiety disorders (such as post traumatic stress disorder) stem from dysregulated fear memory. The excitability of glutamatergic neurons in the BLA correlates with fear memory, and the afterhyperpolarization current (I_AHP_) mediated by small‐conductance calcium‐activated potassium channel subtype 2 (SK2) dominates the excitability of glutamatergicneurons. This study aimed to explore the effect of MPP2 interacts with SK2 in the excitability of glutamatergic neurons in the BLA and the extinction of conditioned fear in mice.

**Methods:**

Fear memory was analyzed via freezing percentage. Western blotting and fluorescence quantitative PCR were used to determine the expression of protein and mRNA respectively. Electrophysiology was employed to measure the excitability of glutamatergic neurons and I_AHP_.

**Results:**

Fear conditioning decreased the levels of synaptic SK2 channels in the BLA, which were restored following fear extinction. Notably, reduced expression of synaptic SK2 channels in the BLA during fear conditioning was caused by the increased activity of protein kinase A (PKA), while increased levels of synaptic SK2 channels in the BLA during fear extinction were mediated by interactions with membrane‐palmitoylated protein 2 (MPP2).

**Conclusions:**

Our results revealed that MPP2 interacts with the SK2 channels and rescues the excitability of glutamatergic neurons by increasing the expression of synaptic SK2 channels in the BLA to promote the normalization of anxiety disorders and provide a new direction for the treatment.

## Graphical abstract

Reduced expression of synaptic SK2 channels in the BLA during fear conditioning was caused by the increased activity of protein kinase A (PKA), while increased levers of synaptic SK2 channels in the BLA during fear extinction were mediated by interactions with membrane‐palmitoylated protein 2 (MPP2).

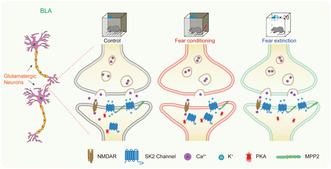

## 1 INTRODUCTION

Amygdala is an almond‐shaped cluster of nuclei located deeply and medially within the temporal lobes of the brain in complex vertebrates and modulates emotion, mood, memory, and cognition. The basolateral amygdala (BLA), which is divided into the lateral amygdala (LA) and basal amygdala (BA), as one of the main component of the amygdala, plays an important role in the regulation of emotion, memory, and anxiety disorders.^1^, ^2^, ^3^ Altered the excitability of glutamatergic neurons in BLA is surveyed in the pathophysiology of anxiety, depression, memory impairments, and post‐traumatic stress disorders (PTSDs).^4^, ^5^ Fear memory has attracted much attention because of its close relationship with psychological trauma. In recent years, fear conditioning and extinction have been widely used to assess fear memory, in which foot shock is considered a kind of traumatic stress to acquire fear.^6^, ^7^ Understanding the factors that reduce fear memory may help patients with emotional disorders recover their normal lives and reduce the social medical burden. The mechanism of fear extinction is still unclear.

SK2 channels are well known for their role in glutamatergic function and reducing excitatory drive by augmenting the afterhyperpolarization phase of action potential.^8^, ^9^ SK2 channels are one type of SK channel, a group of potassium channels with a conductivity value of only 10–20 ps. Immunohistochemical results showed that the SK2 channel protein was mainly expressed in the BLA, but SK1 and SK3 were almost undetectable.^10^ Ultrastructural analysis showed that SK2 channels localize to the postsynaptic density (PSD) of neuronal dendritic spines.^11^, ^12^ The SK2 channel specifically mediates medium hyperpolarizing potential (mAHP), which reduces the excitability of neurons through a decrease in the firing rate.^13^, ^14^

The specific location of the SK2 channel along the neuronal surface is associated with the regulation of mAHP; in addition, mAHP is almost completely produced by postsynaptic mechanisms.^15^, ^16^ Cytoplasmic protein kinase A (PKA) regulates cell signaling and the function of neurons by phosphorylating ion channels. The subcellular distribution and function of the SK2 channel are affected by direct PKA‐mediated phosphorylation. PKA‐mediated phosphorylation of three serine residues (Ser568–570) in the C‐terminal domain of the SK2 channel leads to endocytosis of the synaptic SK2 channel.^17^, ^18^ PKA attenuates mAHP, which is specifically mediated by the SK2 channel, by reducing the expression of the SK2 channel at synapses.

Membrane palmitoylated protein 2 (MPP2/p55) is a member of the MAGUK family. Membrane‐associated guanylate kinases (MAGUKs) are categorized as scaffold proteins that have many roles in regulating synaptic function and structure and the location and function of synaptic receptors. A recent study indicated that MPP2 and SK2 channels are highly co‐expressed in the PSD of dendritic spines on hippocampal CA1 pyramidal neurons. A complex of the SK2 channel (N‐terminal) and MPP2 (SH3‐GK domain) in the brains of mice was identified. The SK2 channel contribution to synaptic responses was abolished by downregulating MPP2 expression. In addition, the knockdown of MPP2 expression decreased LTP.^19^

Here, we used paired CS–US and CS alone (unpaired US) to establish the models of conditioned fear memory conditioning and extinction. We hypothesized that PKA phosphorylates the SK2 channel, resulting in endocytosis of the SK2 channel and a decrease in its expression at the synaptic, which promotes the fear response. During fear extinction, the expression and function of synaptic MPP2 increase, resulting in an increase in the number of postsynaptic SK2 channels, which inhibit the fear response. By studying the expression, distribution, and molecular mechanism of the SK2 channel in the process of fear memory, the results will clarify that the modulation of the SK2 channel is an important neurobiological mechanism for fear memory in individuals with PTSD and provide a target for clinical translation.

## 2 MATERIALS AND METHODS

### 2.1 Animal care

Adult male C57BL/6J mice (8 weeks) were purchased from the Jinan Pengyue Laboratory Animal Breeding Co. Ltd. (Jinan, Shandong, China). Mice had ad libitum access to food and water. The room temperature was 23 ± 1°C, and the light/dark environment was 12 h/12 h. All the experiments were approved by the Institutional Animal Care and Use Committee of Xuzhou Medical University and fully compliant with the National Institutes of Health Guidelines for the Use and Care of Laboratory Animals. This study was approved by the Xuzhou Medical University experimental animal ethics committee.

### 2.2 Delay fear conditioning and extinction

**FIGURE 1:**
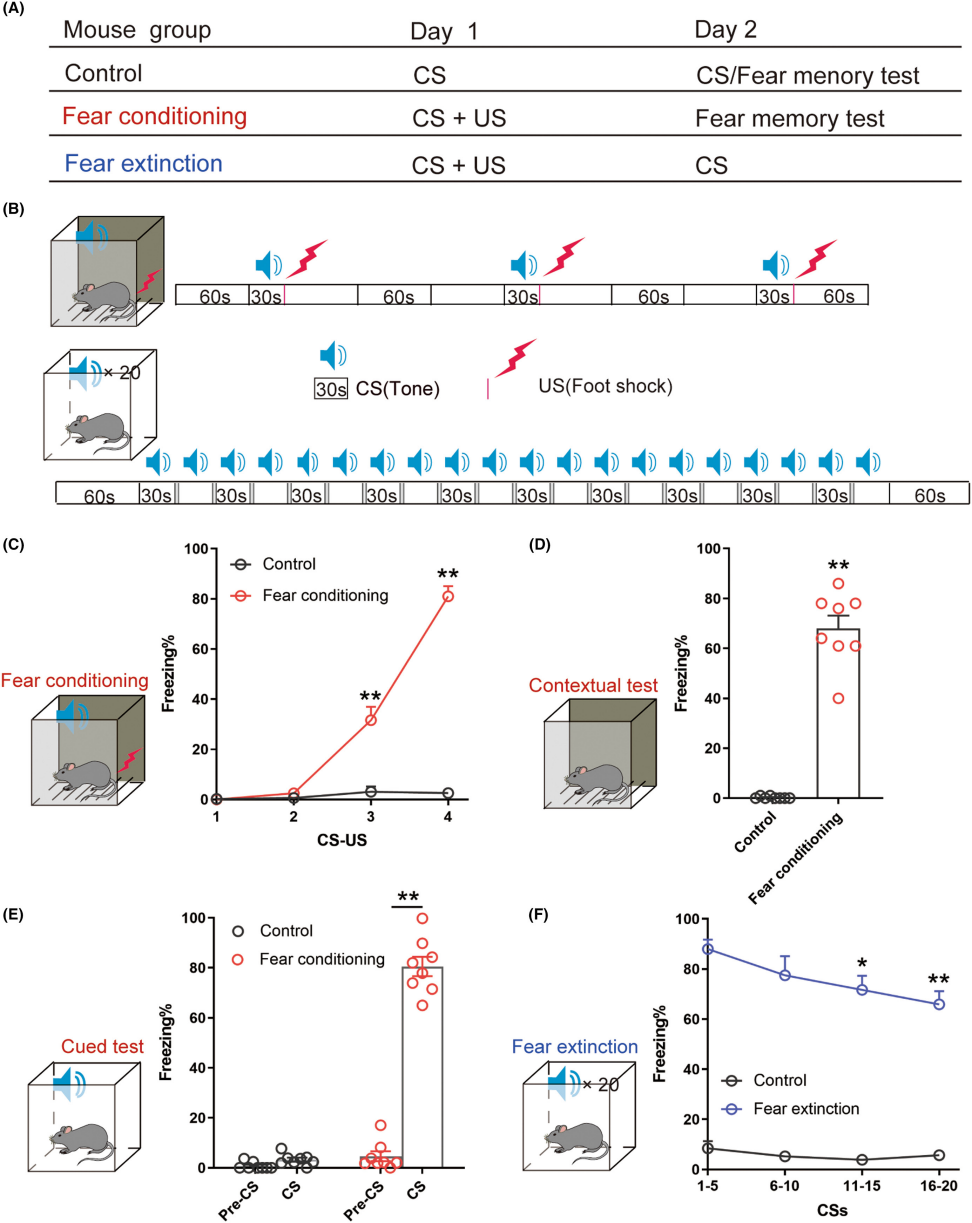
Fear conditioning and extinction in cue‐dependent fear conditioning paradigms in mice. (A,B) Schematic diagram of the Control, Fear conditioning and extinction groups. (C) The percentage of freezing in mice during cued fear memory test (Mann–Whitney test, *p* = 0.000155). (D) The percentage of freezing in mice during fear acquisition (two‐way ANOVA, *p* < 0.01). (E) The percentage of freezing in mice during the contextual fear memory test (Mann–Whitney test, *p* = 0.0002). (F) The percentage of freezing in mice during fear extinction (two‐way ANOVA, *p* = 0.0293). *n* = 8. *p < 0.05, **p < 0.01.

The conditioned fear box had two different contexts, context A and context B. Context A had black polyester walls and a stainless‐steel fence floor. Context B (different backgrounds, smell, and light) had white polyester fiber walls and a smooth white polyester fiber floor. Behavioral procedures were performed and improved (foot shock of 1 mA) based on previously described methods by another lab.^20^ After a day of habituation, 30 min later each mouse was permitted to freely explore Context A during a 10‐min context pre‐exposure session. On the second day, after 30 min in the behavioral laboratory, each mouse was put into context A for three pairs of CS (sound stimulation, 30S, 90Db, 8000 Hz)–US (foot shock, 1 s, 1 mA). On the following day, each mouse was placed in context B for 20 times CS, these mice were fear extinction group. The mice only received three pairs of CS–US were fear conditioning group, and the mice in the control group only received CS (Figure 1A). We used the percentage of freezing to represent the fear response to evaluate fear memory. The freezing behavior was digitally scored and all testing was performed using the Near Infrared Fear Conditioning System (Med Associates, St. Albans, VT).

### 2.3 Determination of freezing behavior

After 24 h of fear conditioning, the mice were tested for their fear response, which was freezing behavior. The mice were placed in the behavioral laboratory for adaptation for 30 min, and then placed in context A for 5 min without (any) tone, and the freezing time was observed to test the mouse fear responses as the contextual fear memory test. Before the test, 75% alcohol was used to clean the test box. Two hours later, the mice were placed in context B to freely explore context B 60 s, then received CS (30 s) for the observation of their freezing behavior, which was used as a cued test of fear memory. Before the test, the test box was cleaned with 10% acetic acid. The freezing behavior was also digitally scored and performed using the Near Infrared Fear Conditioning System (Med Associates, St. Albans, VT).

### 2.4 Immunofluorescence staining

Male mice were deeply anesthetized followed by serial intracardial perfusions with 0.9% saline (100 mL/100 g), and 4% polyformaldehyde in phosphate buffer (100 mL/100 g). Whole brains were rapidly dissected and postfixed with 4% polyformaldehyde at 4°C overnight and cryoprotected with 30% sucrose at 4°C. Then, the brain tissues were embedded, frozen in OCT at −20°C, and sectioned into 30‐μm slices using a Leica microtome. Brain sections were permeabilized with 0.1% Triton X‐100 in PBS, blocked with 5% normal bovine serum in PBS, and incubated with primary antibodies anti‐CaMKII‐ɑ (1:1000, Invitrogen, USA), anti‐c‐Fos (1:1000, Abcam, USA) or anti‐SK2 (1:100, Alomone labs, Israel) at 4°C overnight. Subsequently, the brain slices were rinsed three times, incubated with an Alexa Fluor 488‐conjugated anti‐rabbit (1:1000, Invitrogen, USA) or Alexa Fluor 594‐conjugated anti‐mouse antibody (1:1000, Invitrogen, USA) at r/t for 2 h, and mounted on positively charged slides. Images were obtained with a confocal laser scanning microscope.

### 2.5 Chemogenetic tagging of glutamatergic neurons in the BLA region

Glutamatergic neurons in the BLA region were labeled with rAVV‐CaMKIIα‐hM4D(Gi)‐mCherry‐WPRE‐pA (BrainVTA, China) or rAVV‐CaMKIIα‐mCherry‐WPRE‐pA. hM4D(Gi) (BrainVTA, China) was activated by the selective ligand clozapine‐N‐oxide (CNO, i.p.), which was dissolved in 2% DMSO and diluted in sterile saline to a final injection volume of 0.3 mL/kg. Mice undergoing administration of CNO were assessed for fear conditioning or extinction 60 min after injection.

### 2.6 Optogenetic tagging of glutamatergic neurons in the BLA region

For in vivo optogenetic labeling of glutamatergic neurons in the BLA region, mice underwent a bilateral injection of rAVV‐CaMKIIα‐eNpHR3.0‐mCherry‐WPRE‐hGH‐pA (BrainVTA, China) or rAVV‐CaMKIIα‐mCherry‐WPRE‐hGH‐pA (BrainVTA, China) in the BLA. Three weeks later, for photoinhibition, light trains (589‐nm laser, fear conditioning: duration 90 s, interval 60 s; fear extinction: duration of 20 s, interval of 15 s) (YL589T6–100FC, Lasercentury, China) were applied to the mice during fear conditioning or extinction. Photostimulation was adjusted by a stimulus generator (QAXK‐TTL‐MC, Thinkertech, China), and the cables were covered with aluminum foil to prevent light leakage.

### 2.7 Western blotting analysis

The BLA was prepared for western blotting analysis using Syn‐PER Synaptic Protein Extraction Reagent (Thermo Fisher, USA), resulting in both a functional synaptosome fraction and a cytosolic fraction.^21^ The protein concentration was determined by using BCA. SDS–PAGE gels were used to separate the same amounts of proteins before transfer to PVDF membranes. The PVDF membrane was blocked with 5% skim milk or 3% BSA (bovine serum albumin) at room temperature for 2 h, and then incubated overnight at 4°C with anti‐SK2 (1:500; Alomone labs, Israel), anti‐PKA (1:1000 Abcam ab38949), anti‐pPKA (phospho S96, 1:1000 Abcam ab32390), anti‐MPP2 (1:1000; Abcam, USA), anti‐GAPDH (1:1000), or anti‐β actin (1:3000) primary antibodies. After washing with TBST, the PVDF membrane was incubated with an HRP‐conjugated secondary antibody (1:1000) for 45 min at room temperature. Protein bands were illuminated using the BeyoECL Moon kit and captured using the Image ProPlus image analysis system (Media Cybernetics, Inc., Rockville, MD, United States).

### 2.8 Electrophysiological recording

After injecting rAAV‐CaMKIIa‐mCherry‐WPRE‐pA (BrainVTA, China) into the BLA, the mice were fed normally for 3 weeks. One hour after fear conditioning or extinction, 10% chloral hydrate (300 mg/kg) was intraperitoneally injected, and 20 mL of precooled high sugar slicing liquid was injected with a 20 mL syringe for perfusion. After perfusion, the brain tissue of the mice was quickly removed, and the whole brain tissue was immersed in the precooled high sugar slicing solution for 2–3 min. The brain slices containing the BLA area were incubated in a 34°C high sugar solution for 1 h at room temperature for 30 min and then transferred to the perfusion tank of the electrophysiological operation platform for recording. As per our previously published methods, the whole‐cell recording was used to observe stepped firing rates, electrophysiological patch clamp was performed to record the changes of I_AHP_ on glutamatergic pyramidal neurons in BLA.^22^

The pyramidal neurons which we recorded had a pyramidal‐like firing pattern with strong spike‐frequency adaptation and a prominent AHP. Cells were held at −60 mV current‐clamp mode and action potential firings injected by depolarizing current pulse were recorded by the patch‐clamp amplifier. Miniature excitability postsynaptic currents (mEPSCs) were recorded in the presence of tetrodotoxin (TTX, 1 mM) and at −70 mV current clamp mode. To record I_AHP_ of SK channels, held glutamatergic neurons in voltage‐clamp with holding potential of −60 mV and 100 ms depolarizing pulse to 60 mV to evoke an outward current. All records were collected using MultiClamp700b, a patch clamp amplifier, and converted with Digidata1440A. The collected signals were recorded and analyzed using pCLAMP software.

### 2.9 RNA extraction and fluorescence quantitative PCR

Mice were sacrificed approximately 45 min after fear conditioning or extinction, and total RNA was extracted from the BLA with a Spin Column Animal Total RNA Purification Kit (Sheng‐gong, China) according to the manufacturer's instructions. RNA preparations were reverse transcribed to generate cDNAs. The cDNA products were used as templates for real‐time PCR analysis to measure SK2 or MPP2 expression using SK2‐specific primers (Sheng‐gong, China) or MPP2‐specific primers (Sheng‐gong, China). Sense and antisense primers were selected to be located on different exons to avoid false‐positive results caused by genomic DNA contamination. PCR was performed with a Light Cycler 480 System using fluorescent SYBR Green technology (Applied Biosystems). Reaction protocols had the following format: 1 min at 95°C for enzyme activation followed by 40 cycles of 10 s at 95°C, 30 s at 60°C and 30 s at 72°C. Then, the samples were incubated at 95°C for 5 s and 60°C for 60 s. A melting curve analysis was performed to assess the specificity of the amplification products. All reactions contained the same amount of cDNA templates. The relative expression ratio of SK2 mRNA was normalized to GAPDH gene expression using the △Ct method (2^−△△Ct^). The primer sequences (Sheng‐gong, China) were as follows (5′‐3′, F: forward, R: reverse):SK2:F:*CTGCTTGCTTACTGGAATCATG*, R:*CATCATGAAATTGTGCACATGC*; MPP2:F:*TGTGCGTGCTGGATGTCAACC*, R:*GATCTGCCTCCGTCAACTGCTTAG*; GAPDH:F:*AGGCCGGTGCTGAGTATGTC*, R:*TGCCTGCTTCACCACCTTCT*.

### 2.10 Stereotactic microinjection

We anesthetized the mouse with 1.5% isoflurane and implanted a stainless‐steel guide (outer tube) into the BLA nucleus (anteroposterior: 3.14 mm; lateral: −1.46 mm; ventral: −5 mm) at least one week before drugs injection. The injection needle tube (inner tube) was connected to the Hamilton microinjector driven by the/microinjection pump (KD Scientific) through the polyethylene tube. Microinjection was performed in a 0.5 μL volume delivered over 1 min. Anesthesia was stopped immediately after the microinjection. Before removal, the injection needle was left in the original position for an additional 5–10 min to minimize the dragging of the injected liquid along the injection tract. After approximately 30 min, the mice were fully awake, and the next experimental operation was carried out.

### 2.11 Co‐immunoprecipitation

The BLA was extracted and lysed with IP lysis buffer (Beyotime, China). The lysate was centrifuged at 13,000 rpm for 10 min at 4°C. The supernatant was incubated with the indicated anti‐SK2 antibody (1:50, Alomone labs, Israel) overnight at 4°C with rotation. Then, 30 μL of Protein A/G agarose beads (Thermo Scientific) were added and incubated for 3 h. The beads were washed three times with IP lysis buffer; then, 20 μL of 2X SDS loading buffer was added and boiled for 10 min. Bound and eluted proteins were subsequently separated on SDS–PAGE gels and transferred to PVDF membranes. After blocking with 5% skim milk or 3% BSA for 2 h, the membrane was probed with the anti‐MPP2 antibody (1:1000; Abcam, USA) overnight at 4°C. An HRP‐conjugated secondary antibody was applied for 45 min. Blots were detected with the BeyoECL Moon kit and developed with an Image ProPlus image analysis system.

### 2.12 Drug treatment

1‐EBIO (1‐Ethylbenzimidazolinone, 300 ng 1.85 nmol 300 nL) was dissolved in dimethyl sulfoxide (0.4% DMSO, 300 nL). Apamin (2.5 pmol, 300 nL), 8‐Br‐cAMP (8‐Bromoadenosine 3′, 5′‐cyclic monophosphate, 2.5 pmol, 300 nL), and Rp‐8‐Br‐cAMP (30 pmol, 300 nL) were dissolved in saline. siRNA (siRNA targeting MPP2, sense5′‐*CCUAGAACAUGGCGAGUAUTT*‐3′, antisense5′‐*AUACUCGCCAUGUUCUAGGTT*‐3′, 1 μg/μL, 300 nL), NC (negative control [NC], sense 5′‐*UUCUCCGAACGUGUCACGUTT*‐3′ antisense 5′‐*ACGUGACACGUUCGGAGAATT*‐3′, 1 μg/μL, 300 nL) was designed and produced by Suzhou Genepharma Co., Ltd. Controls were administered the vehicle alone.

### 2.13 Statistics

The Shapiro–Wilk test was used to check whether the data were normally distributed. One sample *t*‐test, Student's *t*‐test (two groups), one‐way ANOVA and two‐way ANOVA were used to analyze the data which have a normal distribution. Data that do not have normal distribution were analyzed via the Kruskal–Wallis test (two groups) and the Mann–Whitney test. SPSS19 and GraphPad Prism 9.0 software were used to analyze the data and generate graphs. All the data are presented as mean ± SEM. *p* < 0.05 was considered significant.

## 3 RESULTS

### 3.1 Fear conditioning and extinction in cue‐dependent fear conditioning paradigms in mice

We used fear conditioning and extinction to model the acquisition and extinction of fear memory in anxiety disorders. On Day 1, the mice received three paired CS‐US as fear conditioning in the fear box where the tone (30 s, 90 dB, 8000 Hz) served as the CS and foot shock (1 s, 1 mA) served as the US. On Day 2, mice were returned to the other box (a modified chamber with different colors, context, lighting, and odors) for 20 CSs for fear of extinction. Cued and contextual fear memory were tested on the second day of fear acquisition (Figure 1A,B). From the second CS–US, the percentage of freezing in the Fear conditioning group was higher than that in the Control group, and as the number of CS–US increased, the percentage of freezing increased significantly (Figure 1C). Through the contextual fear memory (Figure 1D) and cued fear memory tests (Figure 1E) we found that the freezing time of mice in the Fear conditioning group was higher than that of the Control group. When fear extinction occurred, the percentage of freezing decreased with the increase in the number of CSs, and the difference was statistically significant from 11 to 20 CSs (Figure 1F). Moreover, we measured the anxiety‐like and depression‐like behavior and recognition ability of the three groups of mice through behavioral experiments. The results showed that the anxiety‐like and depression‐like behavior of the mice in the Fear conditioning group was stronger than that of the Fear extinction group and the Control group, while recognition ability was less than that of the other two groups (Supporting Information Figure S1). In conclusion, we concluded that CS–US induced fear memory in mice, while repeated exposure to multiple CSs alone induced fear extinction on the second day.

### 3.2 Glutamatergic neurons in the BLA are activated during fear conditioning and extinction

**FIGURE 2:**
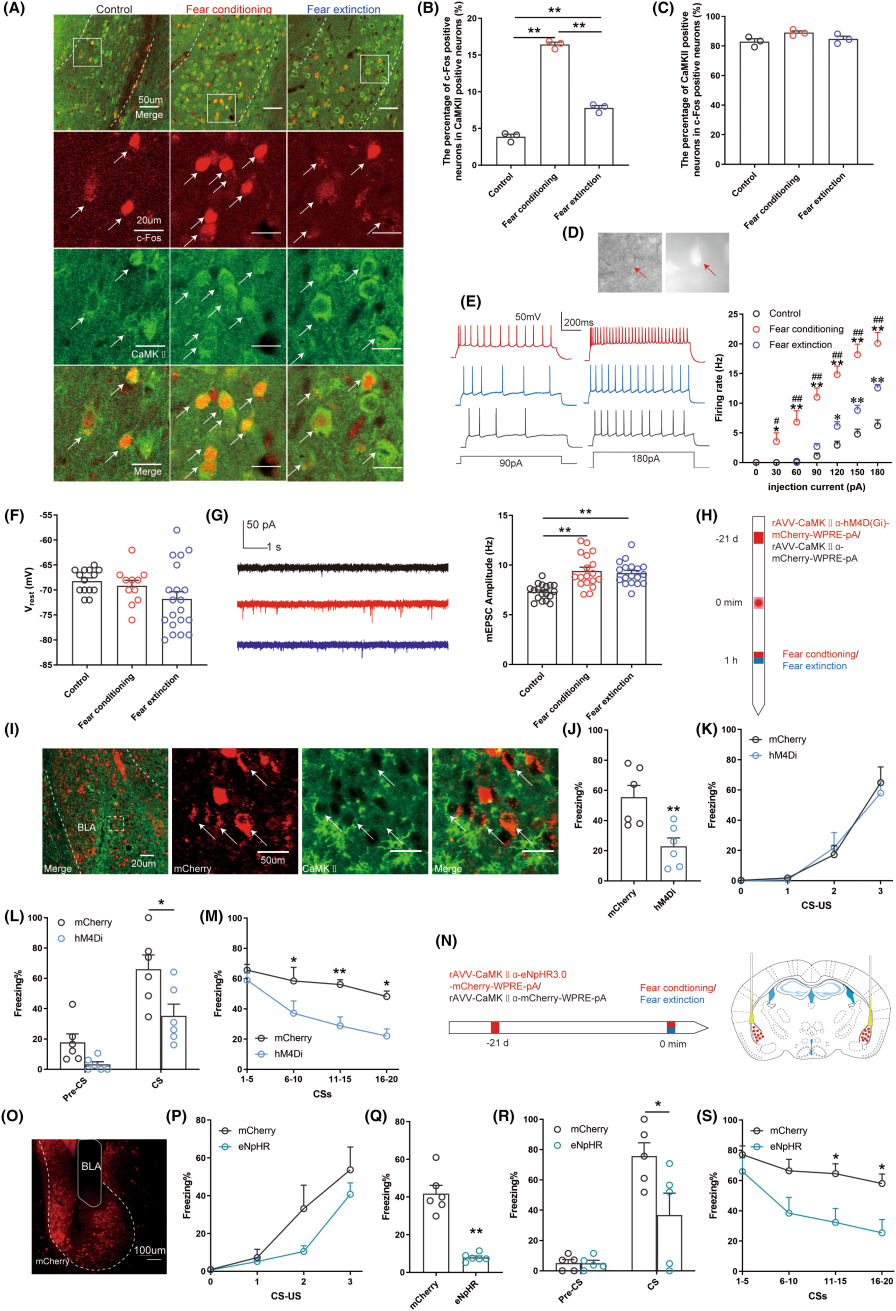
Activation of glutamatergic neurons in the BLA of mice during fear conditioning and extinction. (A) Representative images showing the expression of c‐Fos and CaMKII in the BLA. (B) Quantification of the c‐Fos^+^ proportion among CaMKII^+^ neurons (one‐way ANOVA, *p* < 0.01, *n* = 3). (C) Quantification of the CaMKII^+^ proportion among c‐Fos^+^ neurons (one‐way ANOVA, *p* = 0.1093, *n* = 3). (D) The red arrow indicates neurons that express the virus. (E) The evoked firing rate of glutamatergic neurons in the BLA was increased in a stepwise manner with the stimulus cascade (0–180 pA) (two‐way ANOVA, *p < 0.05, **p < 0.01_Fear conditioning group versus Control group_, ^#^ *p* < 0.05 ^##^ *p* < 0.01_Fear conditioning group versus Fear extinction group_; *p < 0.05, **p < 0.01_Fear extinction group versus Control group_, *n* = 11–20 cells, 5 mice/group). (F) Statistical data of the resting membrane potential as indicated in (E) (one‐way ANOVA, *p* = 0.0971). (G) Representative traces and summary data showing miniature excitability postsynaptic currents in glutamatergic neurons (one‐way ANOVA, *p* < 0.0001, *n* = 18 cells, 3 mice/group). (H) Experimental protocol of chemogenetic manipulation. (I) Representative images showing the expression of mCherry and CaMKII in BLA neurons. (J) The percentage of freezing in mice during contextual fear memory test (unpaired *t*‐test, *p* = 0.0066, *n* = 6). (K) The percentage of freezing in mice during fear acquisition (two‐way ANOVA, *p* = 0.8342, *n* = 6). (L) The percentage of freezing in mice during the cued fear memory test (one‐way ANOVA, *p* = 0.0205, *n* = 6). (M) The percentage of freezing in mice during fear extinction (two‐way ANOVA, *p* < 0.0001, *n* = 6). (N) Experimental protocol of optogenetic manipulation. (O) Representative image illustrating eNpHR3.0‐mCherry expression and electrode placement in the BLA. (P) The percentage of freezing of mice in mice during fear acquisition (two‐way ANOVA, *p* = 0.055, *n* = 6). (Q) The percentage of freezing of mice in mice during the contextual fear memory test (unpaired *t‐*test, *p* < 0.0001, *n* = 6). (R) The percentage of freezing in mice during the cued fear memory test (one‐way ANOVA, *p* = 0.026, *n* = 5). (S) The percentage of freezing in mice during fear extinction (two‐way ANOVA, *p* < 0.0001, *n* = 6). *p < 0.05, **p < 0.01.

We observed the excitability of glutamatergic neurons in the BLA during fear conditioning and extinction by performing immunofluorescence staining and recording the evoked firing rate and miniature excitability postsynaptic currents. First, we observed the co‐expression of c‐Fos and CaMAKII protein using immunofluorescence dual‐labeling. The co‐expression of c‐Fos and CaMKIIα proteins was higher in the Fear extinction group than that in the Control group and lower than that in the Fear conditioning group, indicating that the excitability of glutamatergic neurons was increased through fear conditioning and restored during fear extinction (Figure 2A,B). The percentage of CaMKII‐positive neurons among c‐Fos‐positive neurons in the BLA was more than 80%, which indicated that most of the neurons activated by fear conditioning and fear extinction were glutamatergic neurons (Figure 2A,C). mCherry‐labeled glutamatergic neurons were observed in the BLA by microinjecting rAVV‐CaMKIIα‐mCherry‐WPRE‐pA, which contains the CaMKIIα promoter (Figure 2D). The evoked firing rate of glutamatergic neurons was observed using the whole‐cell patch‐clamp technique. With different injection currents, the evoked firing rate of glutamatergic neurons in the BLA of mice in the Fear extinction group was higher than that in the Control group and lower than that in the Fear conditioning group (Figure 2E). No significant differences were found in the passive membrane properties of pyramidal glutamatergic neurons in the three groups of mice (Figure 2F). As shown by whole‐cell recording in brain slices, fear conditioning and extinction elicited miniature excitability postsynaptic currents in glutamatergic neurons (Figure 2G).

We transduced BLA glutamatergic neurons with hM4Di fused to mCherry, or with the same viral vector carrying only mCherry using recombinant adeno‐associated virus under the control of the CaMKIIα promoter to explore the effects of glutamatergic neurons in the BLA on fear memory in mice. CNO (0.3 mg/kg) was intraperitoneally injected to activate the virus 1 h before fear conditioning or extinction (Figure 2H). mCherry‐positive puncta were immunoreactive for anti‐CaMKII (Figure 2I), consistent with the localization of the virally expressed protein to glutamatergic neurons in the BLA. In fear learning, the percentage of freezing time in the hM4Di group was similar to that in the Control group, indicating that chemogenetic inhibition of glutamatergic neurons in the BLA did not affect fear acquisition in mice (Figure 2K). Compared to control subjects, the hM4Di group exhibited a decreased fear response, as indicated by a lower percentage of freezing during contextual and cued fear memory tests and fear extinction (Figure 2J,L,M).

Next, in optogenetic experiments, we microinjected the mouse BLA with viruses expressing either the inhibitory opsin eNpHR3.0 or mCherry control virus under control of the CaMKIIα promoter and observed its role in fear memory (Figure 2N,O). In fear learning, neuronal inhibition induced by eNpHR3.0 did not affect the percentage of the freezing time but decreased the fear response in the contextual and cued fear memory tests and during fear extinction (Figure 2P–S).

In summary, glutamatergic neurons in the BLA were hyperactivated during fear conditioning and this hyperactivation was rescued by fear extinction. Inhibition of this hyperactivation by chemogenetics and optogenetics reduced the fear response in contextual and cued fear memory and during fear extinction in mice.

### 3.3 SK2 channels in glutamatergic neurons of the BLA are associated with fear conditioning and extinction

**FIGURE 3:**
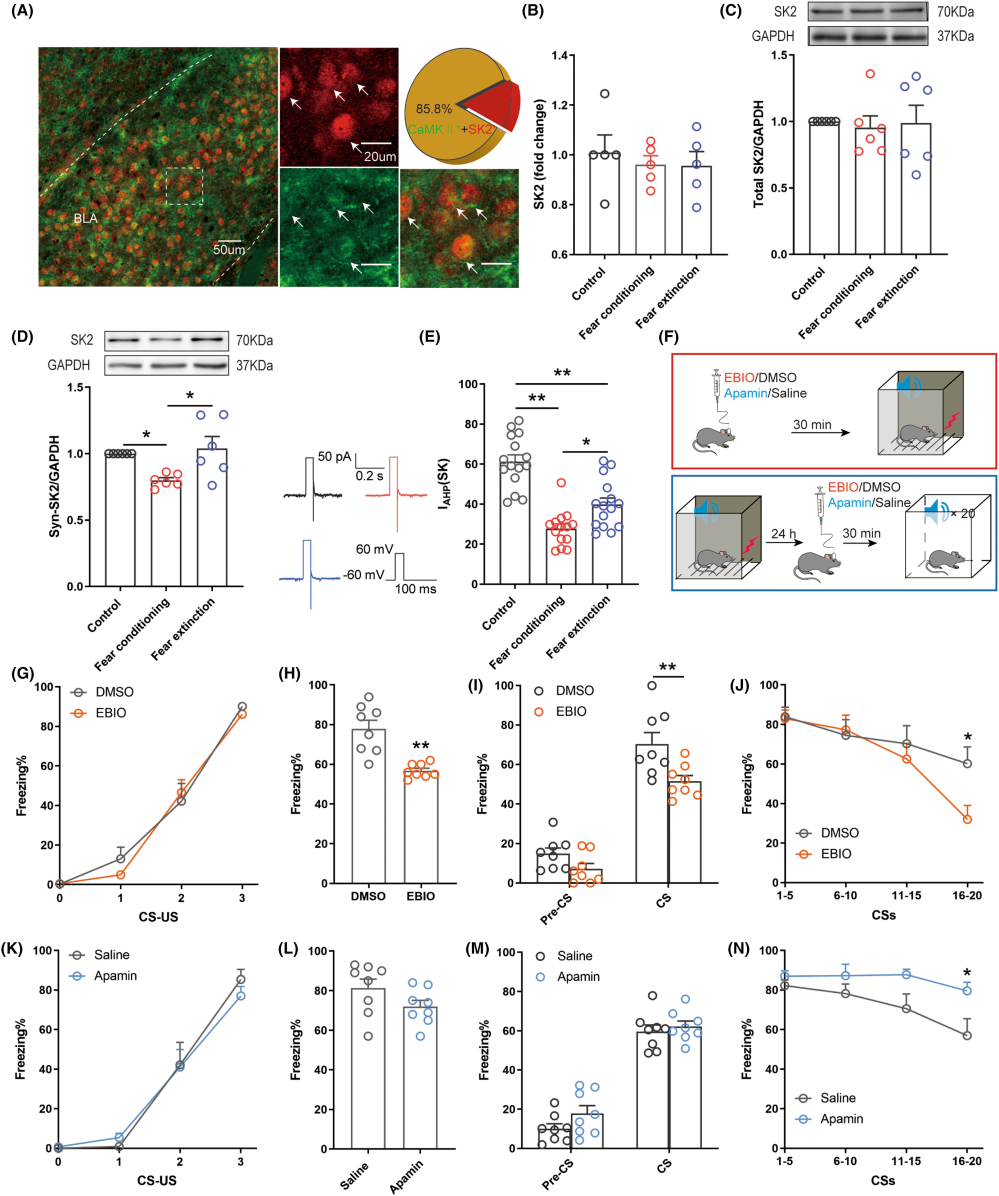
SK2 channels in glutamatergic neurons of the BLA play a role in fear conditioning and extinction. (A) Representative image showing co‐staining with an SK2 antibody and CaMKII antibody in the BLA and quantification of the CaMKII^+^ proportion among SK2^+^ neurons, *n* = 9. (B) The mRNA expression of the total SK2 channel (one‐way ANOVA, *p* = 0.7640, *n* = 5). (C) Western blot analysis of the expression of the total SK2 channel protein in the BLA (Kruskal–Wallis test, *p* = 0.5178, *n* = 6). (D) Western blot analysis of the expression of synaptic SK2 channel proteins in the BLA (the Kruskal–Wallis test, *p* = 0.0070, *n* = 6). (E) The average peak amplitude of I_AHP_ in the Fear conditioning group was reduced compared to that in the other two groups. The average peak amplitude of I_AHP_ in the Fear extinction group was higher than that in the Fear conditioning group but lower than that in the Control group (one‐way ANOVA, *p* < 0.0001, *n* = 14–15 cells, 5 mice/group). (F) Experimental protocol for the pharmacological manipulation. (G,K) The percentage of freezing of mice in the fear acquisition test (two‐way ANOVA, G, *p* = 0.6462; K, *p* = 0.8355, *n* = 8). (H,L) The percentage of freezing of mice during the contextual fear memory test (unpaired *t*‐test, H, *p* = 0.0003; L, *p* = 0.1165, *n* = 8). (I,M) The percentage of freezing of mice during the cued fear memory test (one‐way ANOVA, I, *p* = 0.0087; M, *p* = 0.9358, *n* = 8). (J,N) The percentage of freezing of mice in the fear extinction test (two‐way ANOVA, J, *p* = 0.2755; N, *p* = 0.0307, *n* = 8). *p < 0.05, **p < 0.01.

We first determined the expression of the SK2 channel in BLA glutamatergic neurons by performing immunofluorescence dual‐labeling to investigate the relationship between the SK2 channel and glutamatergic neurons. Quantification of the expression of SK2 and CaMKII in BLA neurons showed that more than 80% of CaMKII‐expressing cells colocalized with SK2^+^ neurons, suggesting that most of the neurons expressing SK2 channel are glutamatergic neurons in the BLA (Figure 3A). Then, we explored the expression of total SK2 channels by performing Western blotting and RT–PCR. Fear conditioning and extinction did not affect the expression of the total SK2 channel (Figure 3B,C). Next, we extracted synaptosome for Western blotting. The expression of synaptic SK2 channels was decreased in mice subjected to fear conditioning and restored in mice in the Fear extinction group (Figure 3D). We proceeded to investigate the relationship between the SK2 channel and the excitability of glutamatergic neurons by recording the I_AHP_ mediated by the SK2 channel expressed on glutamatergic neurons in the BLA. The decrease in the average peak amplitude of I_AHP_ was associated with fear conditioning and the partial increase in this current was related to fear extinction, suggesting that the hyperactivation of glutamatergic neurons correlates with fear conditioning and that fear extinction rescues this hyperactivation (Figure 3E). Taken together, in the BLA, decreasing the expression and function of synaptic SK2 channels is conducive to fear conditioning, while increasing these parameters promotes fear extinction. Then, we observed the effect of SK2 channels on fear conditioning and extinction by regulating them.

The SK2 channel activator 1‐EBIO (300 ng, 300 nL), SK2 channel blocker apamin (2.5 pmol, 300 nL) and their respective controls were microinjected into the BLA 30 min prior to fear conditioning to regulate the SK2 channel and to further explore its role in fear memory (Figure 3F(red)). At 24 h post‐injection, mice were subjected to contextual and cued fear memory tests. Neither 1‐EBIO‐treated mice (Figure 3G) nor apamin‐treated mice (Figure 3K) showed significant differences in fear learning. In the subsequent fear memory test, compared with the DMSO group, the percentage of freezing decreased in the tested mice (Figure 3H,I), suggesting that the contextual and cued fear memory of mice in the 1‐EBIO group were significantly impaired. In contrast, apamin did not exert a significant effect on contextual (Figure 3L) or cued fear memory (Figure 3M).

The mice were randomly divided into 4 groups after fear conditioning to examine the role of the SK2 channel in fear extinction. Then 1‐EBIO, apamin, and their respective controls were microinjected into the BLA 30 min prior to fear extinction. These mice were then subjected to 20 presentations of sound stimulation (CS) in the fear box (Figure 3F (blue)). Compared with the Control group, the percentage of freezing was lower in mice from the 1‐EBIO group, but higher in the apamin group, confirming that 1‐EBIO was helpful for fear extinction (Figure 3J) while apamin prevented fear extinction (Figure 3N). These data indicate that the SK2 channel in the BLA indeed plays a key role in fear conditioning and extinction.

### 3.4 Activated PKA strengthens fear conditioning by phosphorylating synaptic SK2 channels

**FIGURE 4:**
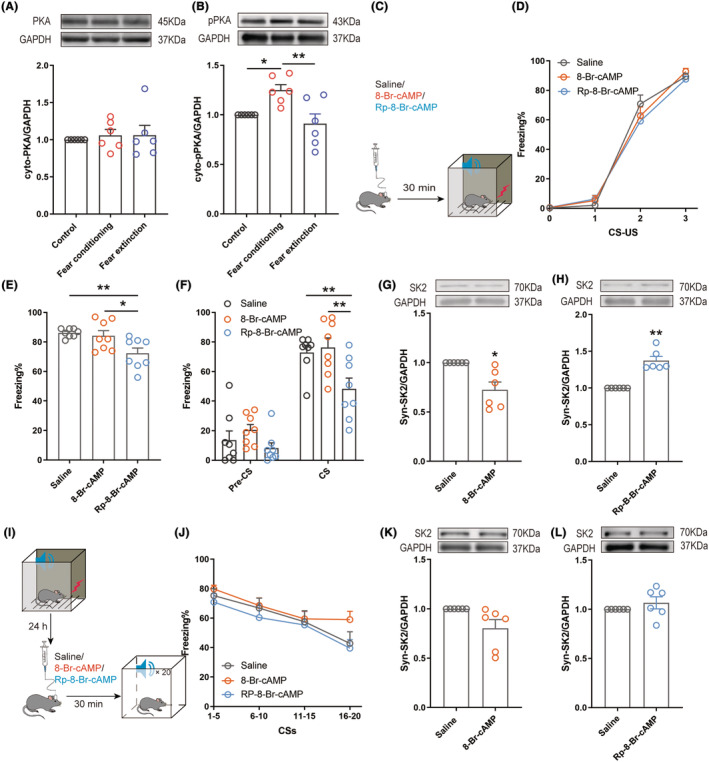
Effects of pharmacological modulation PKA on synaptic SK2 channels to mediate fear conditioning and extinction. (A) Cytoplasmic PKA level in the BLA (the Kruskal–Wallis test, *p* = 0.7492, *n* = 6). (B) Western blot analysis of the expression of cytoplasmic pPKA protein in the BLA (the Kruskal–Wallis test, *p* = 0.0034, *n* = 6). (C,I) Experimental protocol for the PKA manipulation. (D) The fear response of mice in the fear acquisition test (two‐way ANOVA, *p* = 0.6031, *n* = 8). (E) The percentage of freezing of mice in the contextual fear memory test (one way ANOVA, *p* = 0.0068, *n* = 8). (F) The percentage of freezing of mice during the cued fear memory test (one‐way ANOVA, *p* = 0.0020, *n* = 8). (G,H) Western blot analysis of the expression of synaptic SK2 channels (the Mann–Whitney test, G, *p* = 0.0022; H, *p* = 0.0022, *n* = 6) (J) The fear response of mice in the fear extinction test (two‐way ANOVA, *p* = 0.0925, *n* = 8). (K,L) Western blot analysis of the expression of synaptic SK2 channels (one sample *t*‐test, K, *p* = 0.0736, L, *p* = 0.3326, *n* = 6). *p < 0.05, **p < 0.01.

We further explored whether phosphorylated PKA was associated with fear memory by regulating the expression of the SK2 channel. First, we found that fear conditioning and extinction did not affect the expression of cytoplasmic PKA (Figure 4A). However, fear conditioning increased the expression of cytoplasmic pPKA, which was restored by fear extinction (Figure 4B). The PKA activator 8‐Br‐cAMP (2.5 pmol, 300 nL) or inhibitor Rp‐8‐Br‐cAMP (30 pmol, 300 nL) was microinjected into BLA 30 min prior to fear conditioning to determine whether activated PKA reduced synaptic SK2 levels by increasing SK2 phosphorylation (Figure 4C). In fear learning, microinjecting 8‐Br‐cAMP and Rp‐8‐Br‐cAMP into the BLA had no effects on fear conditioning (Figure 4D). In the subsequent contextual and cued fear memory tests, PKA inhibition significantly reduced fear memory, while PKA activation had no effects on fear memory (Figure 4E,F). Next, we obtained BLA tissues 45 min after fear conditioning for Western blotting. Treatment with 8‐Br‐cAMP reduced the expression of synaptic SK2 channels (Figure 4G), and Rp‐8‐Br‐cAMP treatment increased the expression of synaptic SK2 channels (Figure 4H), suggesting that phosphorylated PKA reduced the expression of synaptic SK2 channels during fear conditioning. A potential explanation for this finding is a sufficiently high degree of fear; even if the PKA and synaptic SK2 channels have changed at the molecular level, the behavior cannot express greater fear. In summary, phosphorylated PKA correlated with fear conditioning by reducing the expression of synaptic SK2 channels.

We next examined whether the expression of SK2 mediated by activated PKA was involved in fear extinction. Thirty minutes before fear extinction, 8‐Br‐cAMP, Rp‐8‐Br‐cAMP, or their vehicles were microinjected into BLA of the three groups of mice (Figure 4I). We observed the effect of regulating PKA on fear extinction. The activation or inhibition of PKA did not alter fear extinction (Figure 4J). We obtained BLA tissues 45 min after fear extinction for Western blot analysis. The injection of 8‐Br‐cAMP (Figure 4K) and Rp‐8‐Br‐cAMP (Figure 4L) into the BLA did not affect the expression of synaptic SK2 channels. PKA activation increased the cytoplasmic pPKA level in response to both fear conditioning and fear extinction but did not affect the expression of synaptic SK2 channels. Combined with the molecular results, we speculate that increased expression of SK2 channels by activated PKA may play a major role in fear conditioning, while regulation of PKA to change the expression of synaptic SK2 channels does not play a leading role in fear extinction. What factor plays an important role in mediating the expression of synaptic SK2 during fear extinction?

### 3.5 MPP2 downregulation in the BLA reduced fear extinction by decreasing the expression of synaptic SK2 channels

**FIGURE 5:**
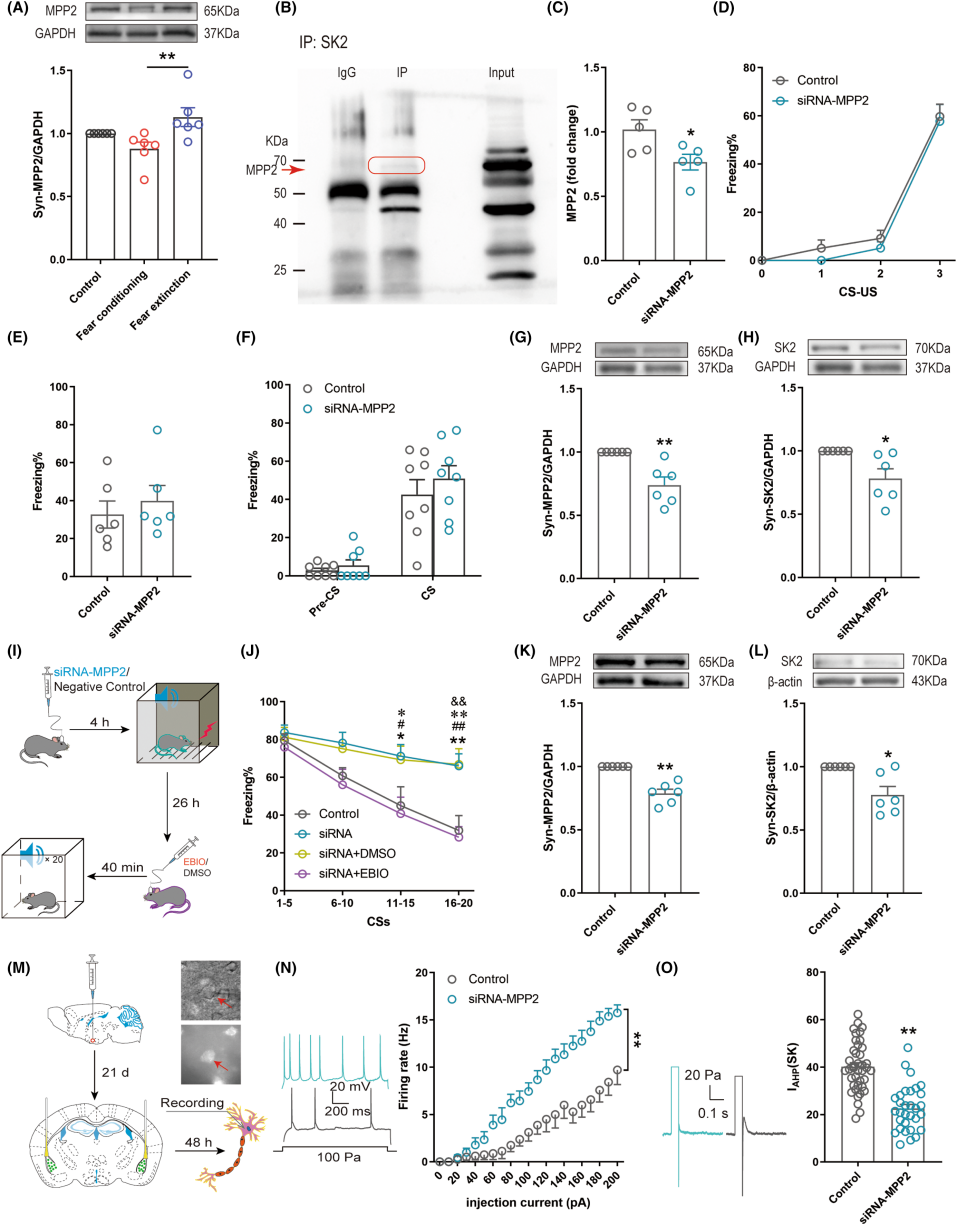
Effects of MPP2 knockdown on synaptic SK2 channels and fear memory. (A) Western blot analysis of the expression of the synaptic MPP2 protein in the BLA (the Kruskal–Wallis test, *p* = 0.0061, *n* = 6). (B) Coimmunoprecipitations: immunoblots prepared from BLA cell lysates expressing SK2, were immunoprecipitated with an anti‐MPP2 antibody or IgG (control). Adjacent blot showing the input of MPP2. (C) The siRNA targeting MPP2 reduced the expression of the MPP2 mRNA (unpaired *t*‐test, *p* = 0.0313, *n* = 5). (D) The fear response of mice in the fear acquisition test (Mann–Whitney test, *p* = 0.853, *n* = 8). (E) The percentage of freezing of mice in the contextual fear memory test (unpaired *t*‐test, *p* = 0.5212, *n* = 6). (F) The percentage of freezing of mice during the cued fear memory test (the Mann–Whitney test, *p* = 0.574, *n* = 8). (G) Western blot analysis of the expression of the synaptic MPP2 protein in the mouse BLA (one sample *t*‐test, *p* = 0.0097, *n* = 6). (H) Western blot analysis of the expression of the synaptic SK2 protein in the mouse BLA (one sample *t*‐test, *p* = 0.037, *n* = 6). (I) Experimental protocol for downregulating MPP2. (J) The percentage of freezing of mice during the fear extinction test (two‐way ANOVA, *p* < 0.01, *p < 0.05, **p < 0.01_Control group versus siRNA group_, *p < 0.05, **p < 0.01_siRNA group versus siRNA + EBIO group_, ^#^ *p* < 0.05, ^##^ *p* < 0.01_siRNA + DMSO group versus siRNA + EBIO group_, ^&&^ *p* < 0.01_Control group versus group_, *n* = 8). (K) Western blot analysis of the expression of the synaptic MPP2 protein in the mouse BLA (one sample *t*‐test, *p* = 0.0014. *n* = 6). (L) Western blot analysis of the expression of the synaptic SK2 protein in the mouse BLA (one sample *t*‐test, *p* = 0.0206; *n* = 6). (M) Schematic showing downregulating MPP2 affects BLA glutamatergic neuron. The red arrow indicates neurons that express the virus. (N) The evoked firing rate of glutamatergic neurons in the BLA was increased in a stepwise manner with the stimulus cascade (0–200 pA) (two‐way ANOVA, *p* < 0.0001, *n*_control_ = 8 cells, *n*_siRNA–MPP2_ = 9 cells, 5 mice/group). (O) The average peak amplitude of I_AHP_ of glutamatergic neurons which were infected by siRNA–MPP2 or NC in BLA (unpaired *t*‐test, *p* < 0.0001, *n*_control_ = 40 cells, *n*_siRNA_–_MPP2_ = 32 cells, 5 mice/group). *p < 0.05, **p < 0.01.

According to previous studies, MPP2 is a synaptic scaffold protein located in the postsynaptic density, and the SK2 channel acts by anchoring to the synapse through MPP2.^19^, ^23^ Therefore, we tested whether synaptic SK2 channels are anchored by the MPP2 scaffold in the BLA. First, in contrast to the mice in the Fear conditioning group, the expression of synaptic MPP2 in the BLA was increased in the Fear extinction group (Figure 5A). We explored whether the SK2 channel interacted with MPP2 in the BLA by performing coimmunoprecipitation experiments. Western blotting with anti‐MPP2 antibody detected a band of the appropriate apparent molecular weight for MPP2 in the BLA sample precipitated with an SK2 antibody, suggesting that the SK2 channel interacts with MPP2 in the BLA (Figure 5B). Next, we used a siRNA targeting MPP2 (siRNA, Gene Pharma, Suzhou, China) to knock down MPP2 expression in the BLA. The PCR results showed that MPP2–siRNA decreased the expression of the MPP2 mRNA (Figure 5C). Thirty hours before fear conditioning, the mice were separately injected with MPP2–siRNA or the NC in the BLA. The percentage of freezing was not significantly different in fear conditioning between the MPP2–siRNA and Control groups (Figure 5D). On the next day, we evaluated the contextual and cued fear memory of mice. The results indicated that MPP2 knockdown had no effect on contextual memory (Figure 5E) or cued memory (Figure 5F). At 45 min after fear conditioning, the BLA was extracted and used for Western blot analysis. The siRNA targeting MPP2 decreased synaptic MPP2 (Figure 5G) and the expression of synaptic SK2 channel levels (Figure 5H) in fear conditioning. In conclusion, knockdown of MPP2 during fear conditioning decreased the expression of synaptic SK2 channel but did not affect fear response.

Four hours before fear conditioning, mice were separately injected with MPP2‐siRNA and NC siRNA in the BLA to examine the effect of MPP2 on fear extinction through the SK2 channel. On the next day, 40 min before fear extinction, the mice in the MPP2‐siRNA group were randomly divided into 3 groups, two of which were injected with 1‐EBIO and DMSO into the BLA (Figure 5I). Compared with control subjects, the siRNA group exhibited increased fear response behaviors, as indicated by a greater number of freezing behaviors during fear extinction, and significant activation of the SK2 channel by 1‐EBIO counteracted the increase in the fear response (Figure 5J). At 45 min after fear conditioning or fear extinction, the BLA was extracted and used for Western blot analysis. The siRNA decreased synaptic MPP2 levels in response to fear extinction (Figure 5K). Microinjection of the siRNA targeting MPP2 into the BLA significantly reduced the expression of synaptic SK2 channels in animals subjected to fear extinction (Figure 5L).

We labeled glutamatergic neurons with the virus rAVV‐CaMKIIα‐mCherry‐WPRE‐pA (Red fluorescence) to test the effect of MPP2 on glutamatergic neurons through the SK2 channel in the BLA. Then, before observing the evoked firing rate of glutamatergic neurons and the average peak amplitude of I_AHP_ through electrophysiology, we injected the siRNA (Green fluorescence) and NC into the BLA (Figure 5M). The evoked firing rate of glutamatergic neurons in the siRNA group was higher than that in the Control group (Figure 5N), and the average peak amplitude of I_AHP_ of glutamatergic neurons in the siRNA group was lower than that in the Control group (Figure 5O). This result suggested that MPP2 knockdown increased the activity of glutamatergic neurons by decreasing the expression and function of synaptic SK2 channels. In conclusion, in the BLA, MPP2 interacts with the SK2 channel, thereby decreasing the activity of glutamatergic neurons to enhance fear extinction, but it does not play a major role in fear conditioning.

## 4 DISCUSSION

In the present study, we provided novel evidence that synaptic SK2 channels in the BLA are involved in the formation and extinction of fear in mice by mediating the excitability of glutamatergic neurons. Specifically, the regulation of synaptic SK2 by PKA participates in the coding of fear memory (CS–US), while MPP2‐mediated changes in synaptic SK2 expression and function play a role in the coding of fear memory extinction (CS‐No US).

The models of fear conditioning and extinction are often used to explore the mechanism of anxiety disorders (such as PTSD). Fear conditioning induced by multiple pairs of CS–US caused mice to acquire associative fear memory. Then, many CS (No US)‐mediated fear extinctions caused the mice to form a new associative memory. Generalization of fear memory and fear extinction disorder are the main symptoms and pathogeny of PTSD. Therefore, studies aiming to discover the factors that enhance or impair fear memory and fear extinction is conducive to exploring the pathogenesis of PTSD and providing new targets for the treatment of PTSD.

The important neural structure involved in PTSD is part of the limbic system, an important region for emotion processing in humans and animals.^24^ In individuals with PTSD, the amygdala, hippocampus and prefrontal cortex are the three most obvious areas of the limbic system shoeing altered function. In studies of Pavlovian conditioned fear in animals and humans, the amygdala regulates fear memory and receives neural projections from the hippocampus and prefrontal cortex.^25^, ^26^, ^27^ In the present study, the excitability of glutamatergic neurons in the BLA was significantly increased during fear conditioning and extinction, while the excitability of glutamatergic neurons in the BLA was lower during fear extinction. Based on these results, glutamatergic neurons in the BLA are hyperactivated during fear conditioning and must be excited to produce associative memory.^28^, ^29^ The explanation for the lower excitability of fear extinction may be due to the inhibitory effect of the new associative memory on fear memory or the low activation of neurons required for the generation of a new associative memory. Inhibiting the excitability of glutamatergic neurons in BLA using chemogenetics and optogenetics weakened cued and contextual fear memory, consistent with the results reported by Sengupta.^30^ Inhibiting the excitability of BLA glutamatergic neurons during fear extinction may cause changes in the excitatory/inhibitory (E/I) balance in the mPFC–BLA pathway, which is beneficial to fear extinction by inhibiting the hyperexcitability of glutamate neurons induced by fear conditioning.^31^

As a key regulator of amygdala neuronal excitability, we speculated that synaptic SK2 channels participate in the regulation of glutamatergic neuronal excitability in the BLA by mediating mAHP. SK channels are located in the postsynaptic membrane of glutamatergic neurons close to N‐methyl‐D‐aspartate receptors (NMDARs), where they are activated by NMDAR‐mediated Ca^2+^ influx. Opening of the SK channel induces K^+^ efflux which alters the membrane potential.^32^ Furthermore, K^+^ efflux and SK channel‐mediated reduction of [Ca^2+^] create a local current that re‐establishes NMDA receptor blockade by Mg^2+^, thereby preventing re‐excitation of the neurons.^33^ The SK2 channel might modulate the excitability of neurons in the BLA by affecting NMDARs. In addition, recent studies have examined the role of SK channels in serotonergic neurons in model systems of mood disorders such as depression.^34^ Further studies are needed to explore how SK2 channels regulate the excitability of neurons in the BLA.

We also explored the mechanisms underlying the roles of synaptic SK2 channels in fear conditioning and extinction. The cytoplasmic pPKA level in the BLA was increased in response to fear conditioning and restored by fear extinction. Then, we perceived that the regulation of PKA was important to the expression of synaptic SK2. Indeed, in fear conditioning PKA activation significantly reduced the synaptic SK2 channel abundance, while PKA inhibition significantly increased the synaptic SK2 channel abundance and fear memory. Therefore, we speculated that PKA activation was involved in the decrease in synaptic SK2 channels in the BLA through phosphorylation and the process of synaptic SK2 channel endocytosis in fear conditioning. As the fear response seems to have reached its peak, further activation of PKA did not significantly increase the fear response. In the BLA, activation of the cAMP–PKA pathway is involved in memory consolidation.^35^, ^36^ Therefore, PKA agonists may activate other pathways (cAMP‐PKA) while promoting the depression of the SK2 channel. Thus, the mechanism by which SK2 mediates fear conditioning is a decrease in the expression of synaptic SK2 induced by cytoplasmic PKA‐mediated phosphorylation. However, the mechanism did not have an important function in fear extinction.

Furthermore, we identified a major role for MPP2 in regulating the expression of synaptic SK2 channels during fear extinction. In the BLA, the SK2 channel interacts with MPP2. Knockdown of MPP2 decreased the expression of synaptic SK2 in the BLA and reduced fear extinction. However, MPP2 knockdown did not significantly affect fear memory during fear conditioning. These results supported the view that fear extinction is a new process of associative memory formation, rather than the destruction of the original fear memory. Numerous studies have highlighted that acute disruption of synaptic MAGUKs dramatically affects NMDA receptor‐ or AMPA receptor‐mediated currents and long‐term potentiation.^37^, ^38^, ^39^, ^40^, ^41^ Therefore, we cannot exclude the possibility that reducing MPP2 levels directly or indirectly affects fear memory through its interactions with other channels. The shared domain of MAGUK proteins is the PDZ domain, which plays an important role in promoting the aggregation and function of various ion channels (such as Ca^2+^ channels and K^+^ channels).^42^ In CA1 hippocampal neurons, the SH3‐HOOK‐GK domain of MPP2 interacts with the N‐terminal domain of SK2.^19^ MAGUKs also contain phosphorylation sites, which can be used as auxiliary peptides to regulate the function and format of gated ion channels by interacting with ion channels.^43^ The domain of MPP2 that interacts with the SK2 channel in the BLA and the mechanism by which MPP2 mediates the expression of the SK2 channel must be explored.

## 5 CONCLUSION

**FIGURE 6:**
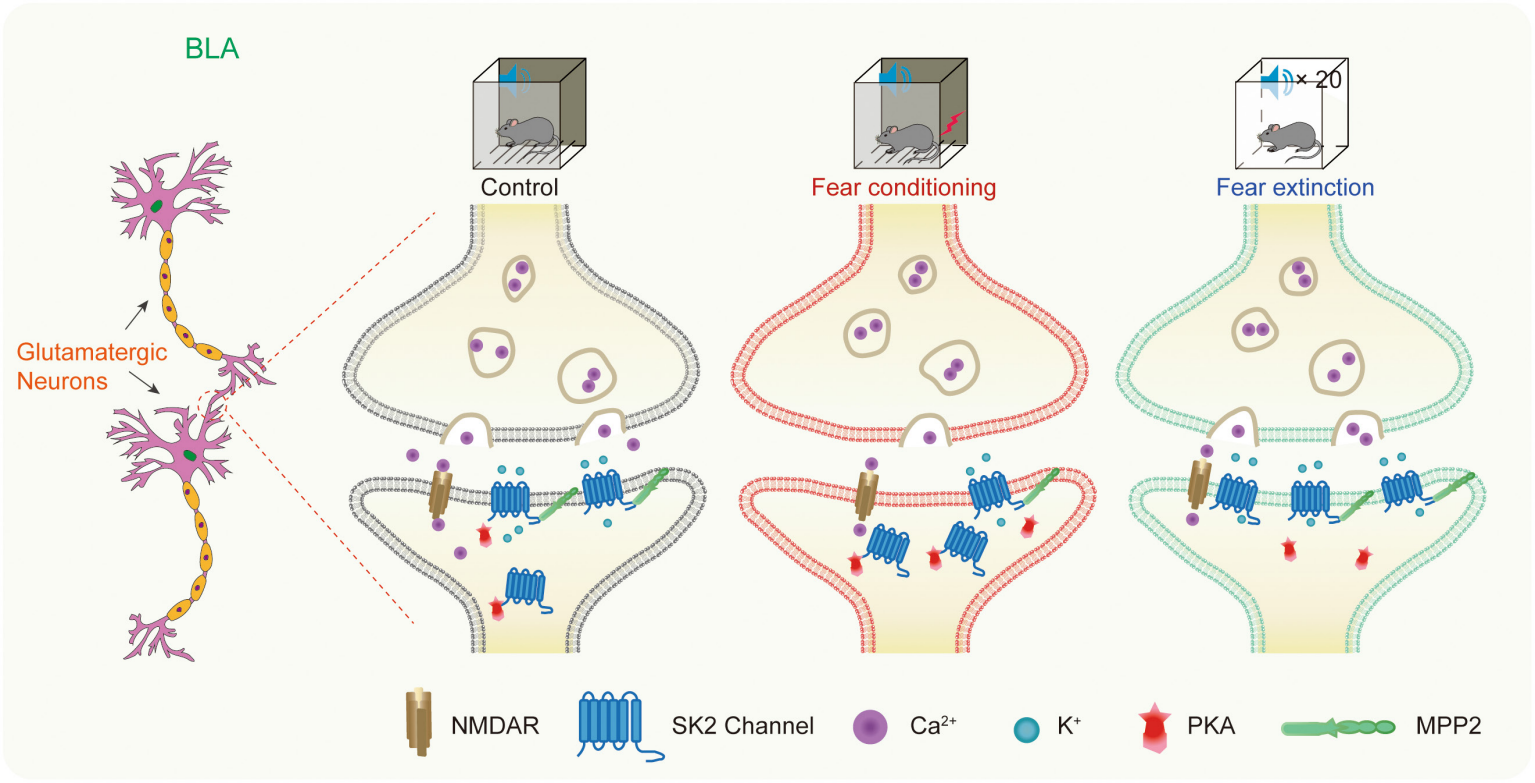
Schematic diagram of the mechanism by which SK2 regulates fear memory.

In conclusion, synaptic SK2 channels in the BLA correlate with the regulation of fear conditioning and extinction by modulating the activity of glutamatergic neurons. In fear conditioning, the excitability of glutamatergic neurons is increased, which may be relevant to activated PKA phosphorylating synaptic SK2 channels, which promotes the endocytosis of the synaptic SK2 channel and reduces its expression in the synapse (Figure 6). Conversely, the decrease in the excitability of glutamatergic neurons might be related to an increase in SK2 channels in the synapse by anchoring the SK2 channel to the synapse through MPP2 during fear extinction (Figure 6). These findings validated that the conditioning and extinction of fear memory are not reversible processes. The mechanism by which MPP2 interacts with SK2 remains to be clarified. Furthermore, studies designed to explore whether MPP2 affects fear memory through other pathways will be important.

## AUTHOR CONTRIBUTIONS

*Conceptualization*: Rong Hua, Yong‐mei Zhang, Tie Xu, Xiaohan Peng, Panpan Chen, and Yang Zhang. *Formal analysis*: Rong Hua, Yong‐mei Zhang, Tie Xu, and Xiaohan Peng. *Funding acquisition*: Rong Hua, Yong‐mei Zhang, and Tie Xu. *Methodology*: Yong‐mei Zhang, Xiaohan Peng, Panpan Chen, Ke Wu, Ningning Ji, Jinghua Gao, and Hui Wang. *Investigation*: Rong Hua, Yong‐mei Zhang, Tie Xu, Xiaohan Peng, Panpan Chen, and Yang Zhang. *Writing—original draft preparation*: Xiaohan Peng, Yang Zhang, Yong‐mei Zhang, Jinghua Gao, and Hui Wang. *Writing—review & editing*: Rong Hua, Yong‐mei Zhang, Tie Xu, Xiaohan Peng, Panpan Chen, Yang Zhang. All the authors have read and agreed to the published version of the manuscript.

## CONFLICT OF INTEREST STATEMENT

The authors declare no conflicts of interest.

## Supporting information


Data S1.

## Data Availability

The data that support the findings of this study are available from the corresponding author upon reasonable request.
