# Supplementary material for: MPP2 interacts with SK2 to rescue the excitability of glutamatergic neurons in the BLA and facilitate the extinction of conditioned fear in mice
Source: CNS Neurosci Ther. 2023 Jul 19;30(1):e14362. doi: 10.1111/cns.14362 (PMC10805397; doi:10.1111/cns.14362)
Supplement: Supplementary file 1 — Data S1. [file CNS-30-e14362-s001.docx]

**Supplement**

**S1 Methods**

**S1.1 Elevated plus maze**

The mice were placed in the center of the elevated plus maze and faced to the closed arms. Their activity within 5 minutes were recorded. Observations included the dwell time and number of times the mice entered the open arms (both front paws must enter the arm). After completing the experiment on each mouse, cleaned the four arms and sprayed 70% alcohol to remove the odor. ANY-maze video tracking software was used for recording.

**S1.2** **Forced Swim Tes**t

Gently placed the mice in a plastic cylinder filled with 20 cm deep water, maintained the temperature at 24 ℃, and observed the immobility time for 6 minutes. The immobility state was defined as the minimum speed required for a mouse to tread on the water while remaining in a stationary position and maintaining its nose and eyes above the water. The first minute was the adaptation period and was not recorded in time.

**S1.3 Open Field Test**

Placed the mice in the center of the open field (50 × 50 cm) and allowed them to explore freely for 5 minutes. During this 5-minute exploration, ANY-maze video tracking software recorded the time that mice staying in the central area (16.6 × 16.6 cm) and the number of times entered the central area.

**S1.4** **Novel Object Recognition**

Novel object recognition was divided into three stages: habit, training, and testing. Habit period, the mice were put into the experimental box (50cm × 50cm × 50cm), explored freely for 10 minutes. Training period, after 2 hours, and placed a green rectangular building block (6cm × 3cm × 3cm) in the same position on both corners of the experimental box, placed the mouse in the center area without facing any objects. The mice were allowed to explore freely for 10 minutes. Test period, after 3 hours, replaced one of the above rectangular blocks with a red cylinder with a diameter of 3 cm and a height of 6 cm. The mice were allowed to explore freely for 10 minutes. The time of they spent exploring two objects was recorded respectively. Touching or sniffing at a distance of less than or equal to 2 cm from an object was considered object exploration. The experimental results were expressed as a discrimination index, which was the percentage between the time spent exploring new objects and the total time spent exploring two objects.

**S1.5 Morris Water Maze**

Morris water maze was divided into two stages: acquired and exploration training. Acquired training, the mice were placed into the water with its head facing the pool wall, and randomly choose one of the four starting positions: east, west, south, and north. Recorded the time when the mice found the underwater platform. In previous training sessions, if this time exceeded 60 seconds, guided the mice to the platform. The mice were allowed to stay on the platform for 10 seconds. Removed and dried the mice and placed the mice under a 150W incandescent lamp for 5 minutes. Each mouse was trained four times a day, with an interval of 15 minutes between the two exercises, for a continuous period of 5 days. Exploration training, the next day after the last acquired training, the platform was removed and 60s exploration training was started. Placed the mice into the water from the opposite side of the original platform quadrant. The time spent by the mice in the target quadrant (the quadrant where the platform was originally placed) and the number of times it entered the quadrant were recorded.

**S2 Result**

In the elevated plus maze, the time for mice in the fear extinction group to enter the open arm was longer than that in the fear conditioning group, with no significant difference compared to the control group (Figure S1B-D). In the open field test, the mice in the fear extinction group entered the central area more often and for longer than those in the fear conditioning group, with no difference compared to the control group (Figure S1E-G). In the forced swim test, the percentage of immobility time of mice in the fear extinction group was significantly lower than that in the fear conditioning group, with no significant difference compared to the control group (Figure S1H). In the novel object recognition, the discrimination index of mice in the fear extinction group was significantly higher than that in the fear conditioning group, which was similar to the control group (Figure S1I). In the Morris water maze, there was no difference among three groups (Figure S1J-L).


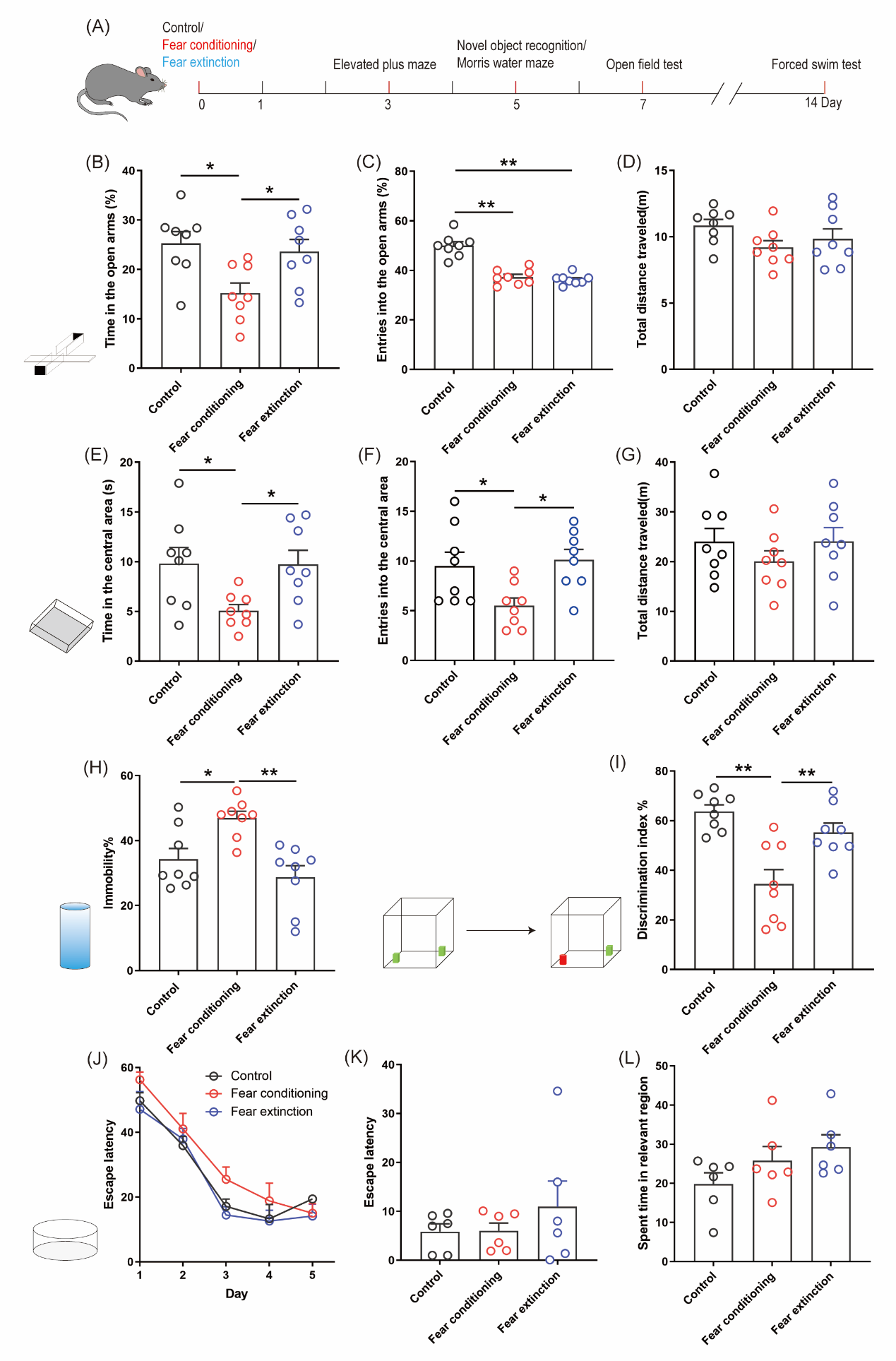


FIGURE S1 Effects of fear memory on anxiety- and depression- like behaviors and recognition ability in mice. (A) Timeline of behavioral testing. (B) The percentage of time that mice entered open arms in the elevated plus maze test (one-way ANOVA, *p* = 0.0016, n = 8). (C) The frequency of entering open arms in the elevated plus maze test (one-way ANOVA, *p* < 0.0001, n = 8). (D) Total travelled distance of mice in the elevated plus maze test (one-way ANOVA, *p* = 0.1617, n = 8). (E) The time of mice staying in central area in the open field test (one-way ANOVA, *p* = 0.0261, n = 8). (G) The number of mice entering the central area in the open field test (one-way ANOVA, *p* = 0.0150, n = 8). (H) Total travelled distance of mice in the open field test (one-way ANOVA, *p* = 0.4561, n = 8). (H) Percentage of immobility time of mice in the forced swim test (one-way ANOVA, *p* =0.0012, n = 8). (I) The discrimination index of mice in the novel object recognition test (one-way ANOVA, *p* = 0.0003, n =8). (J) The escape latency of mice during acquired training period in the marris water maze (two-way ANOVA, *p* = 0.0435, n = 6). (K) The escape latency of mice during exploration training period in the marris water maze (one-way ANOVA, p = 0.4823, n = 6). (L) The spent time in relevant region of mice during exploration training period in the marris water maze (one-way ANOVA, *p* = 0.1448, n = 6). Data are expressed as mean ± SEM. **p* < 0.05, ***p* < 0.01.
